# Alfalfa virus S, a new species in the family *Alphaflexiviridae*

**DOI:** 10.1371/journal.pone.0178222

**Published:** 2017-05-30

**Authors:** Lev G. Nemchinov, Samuel C. Grinstead, Dimitre S. Mollov

**Affiliations:** 1 USDA-ARS, Molecular Plant Pathology Laboratory, Beltsville, Maryland, United States of America; 2 USDA-ARS, National Germplasm Recourses Laboratory, Beltsville, Maryland, United States of America; Oklahoma State University, UNITED STATES

## Abstract

A new species of the family *Alphaflexiviridae* provisionally named alfalfa virus S (AVS) was discovered in alfalfa samples originating from Sudan. A complete nucleotide sequence of the viral genome consisting of 8,349 nucleotides excluding the 3’ poly(A) tail was determined by high throughput sequencing (HTS) on an Illumina platform. NCBI BLAST searches revealed that the virus shares the greatest degree of sequence identity with members of the family *Alphaflexiviridae*, genus *Allexivirus*. The AVS genome contains six computationally-predicted open reading frames (ORF) encoding viral replication protein, triple gene block protein 1 (TGB1), TGB2, TGB3-like protein, unknown 38.4 kDa protein resembling serine-rich 40 kDa protein characteristic for allexiviruses, and coat protein (CP). AVS lacks a clear 3’ proximal ORF that encodes a nucleic acid-binding protein typical for allexiviruses. The identity of the virus was confirmed by RT-PCR with primers derived from the HTS-generated sequence, dot blot hybridization with DIG-labeled virus-specific RNA probes, and Western blot analysis with antibodies produced against a peptide derived from the CP sequence. Transmission electron microscopic observations of the infected tissues showed the presence of filamentous particles similar to allexiviruses in their length and appearance. To the best of our knowledge, this is the first report on the identification of a putative allexivirus in alfalfa (*Medicago sativa*). The genome sequence of AVS has been deposited in NCBI GenBank on 03/02/2016 as accession № KY696659.

## Introduction

Alfalfa (*Medicago sativa*) is the most extensively cultivated forage legume in the world and the fourth most widely grown crop in the US, planted on more than 23 million acres in all 50 states. The estimated worth of alfalfa hay is over $8 billion annually. In addition to its value as livestock forage, alfalfa also has potential to make important contributions to bioenergy production. Alfalfa productivity is often limited by different diseases, insect pests and abiotic stress factors [1,2]. Minimizing these losses is a major area of concern in the alfalfa industry.

Although viral infections of alfalfa are widespread in major cultivation areas, they are generally considered to be diseases of minor or limited importance. The most frequently diagnosed viruses in alfalfa are *Alfalfa mosaic virus* and *Cucumber mosaic virus* [3]. Also, among the different viruses infecting alfalfa are alfalfa enation virus, *Bean leafroll virus*, *Lucerne transient streak virus*, *Pea streak virus*, *Red clover vein mosaic virus*, *Peanut stunt virus*, *Bean common mosaic virus*, and others [4]. Alfalfa may serve as a natural reservoir for dissemination of viruses to other agriculturally important crops, although its exact role in the epidemiology of viruses in other crops is not well documented [5,6]. In recent years, emerging viral diseases of alfalfa with the capacity to cause more serious yield losses, have been described [7,8]. In addition, synergetic interactions of multiple viral infections can threaten alfalfa production [7,9].

In this work, we report identification, complete genomic sequence and specific diagnostics of a novel and unusual virus discovered in alfalfa samples originating from Sudan. The virus, provisionally named alfalfa virus S (AVS), was not previously described in alfalfa or any other plant species.

## Materials and methods

### Plant samples and inoculation of alfalfa plants

Alfalfa samples from four commercial pivot irrigated fields, 60 ha each, in Sudan were submitted for disease analysis. In total, nine different alfalfa samples designated 98.3A, 89.2AS, 89.3H, 89.1H, 89.1AS, 98.1A, 98.3A(0), 98.2RR and 97.2A were used in RT-PCR, dot blot hybridization and Western blot (WB) assays.

### Total RNA extraction, RT-PCR, dot blot hybridization and Illumina RNA sequencing

For dot blot hybridization, RT-PCR assays and for Illumina RNA sequencing, total RNA was extracted from alfalfa samples using TRIzol RNA isolation reagent as described by the manufacturer (ThermoFisher Scientific). For dot blot hybridization, one μl of total RNA (~ 100–300 ng) was dotted directly onto a nylon membrane and cross-linked by ultraviolet light using a UVP HL-2000 HybriLinker (UVP, LLC, CA, USA) for three minutes. Hybridization (performed in UVP HL-2000 HybriLinker hybridization oven), washing and detection conditions were done exactly as described in the Boehringer Mannheim guide for filter hybridization [10].

RT-PCR was performed with total RNA employing SuperScript RT-PCR system according to the manufacturer’s directions (ThermoFisher Scientific). Primers for RT-PCR are shown in Table 1.

**Table 1:** Primers used for RT-PCR.

Gene	Primer name	Primer position	Primer sequence, 5’ to 3’
CP -F	LN411	7170–7190	ATGTCTCAACACCGCACCGAC3
CP-R	LN409	8009–8027	TTATTCTCCAAAGGTGATC
P38.4 kDa-F	LN413	6245–6265	ATGCTTTCAACCGCCAACTTC3’
P38.4 kDa-R	LN414	7260–7279	TTAACACACATTGCTGTGCG
RdRP	LN416	312–332	CTGATGCGTGGGTGTGGCTGG
RdRP	LN423	448–472	GCAGTAGTTGACCAATCTGTCTTGC
RdRP	LN421	289–311	TTGTGGCAAAAGGGGAGGTGGTG3
RdRP	LN422	270–289	GATGCCTAACCGTTCTAGCG3
RdRP	LN424	428–447	CCTGGTCCTCGGTTGAGTAG
3’end- R	LN412	8337–8349	ttttttttttttttttttttttGTTGCGTAGAACC
3’ end-F (CP)	LN427	7955–7976	CCGCGGTAATATGATGGGTATG

Note: F, forward primer; R, reverse primer. CP, coat protein; p38.4kDa, unknown ORF5; RdRP, viral replicase.

RT-PCR products were either sequenced directly or cloned into pCRII-TOPO vector with dual promoter (ThermoFisher Scientific) for sequencing and synthesis of DIG-labeled RNA probes. DIG-labeled probes were synthesized using DIG RNA labeling mixture and T7 RNA polymerase (ThermoFisher Scientific) essentially as described [10].

Library construction and high-throughput sequencing (HTS) on an Illumina Platform were outsourced and performed by SeqMatic Inc. (Fremont, CA). Contigs were assembled from the read data using CLC Genomics Workbench software version 9.5.2.

The 5’end of the virus was determined using the 5-RACE system (ThermoFisher Scientific), PCR with several genome-specific primers (LN416, LN421-424; Table 1) and sequencing. The 3’ end of the genome was obtained by RT-PCR with primers LN412/LN427 and sequencing of the resulting PCR products.

### Western blot analysis

A western blot assay was performed with the original tissues as described in Nemchinov and Natilla [11]. Membranes were probed with affinity-purified polyclonal antibodies (Abs) against three synthetic peptides derived from the coat protein sequence of AVS: CP1, ESNPRVIPPNPNET, aa 49–62 of CP; CP2, GSRTNPHQYSTRGN, aa 262–265; and CP3, ARGETSHRIPLAKI, aa 150–163. Peptide antigens and Abs were produced by GenScript according to the company protocols (GenScript Inc, NJ, USA). Reactions were developed with NBT/BCIP phosphatase substrate (Kirkegaard and Perry Laboratories, Inc., Gaithersburg, MD).

### Transmission electron microscopy

For transmission electron microscopy (TEM), leaves were homogenized in sterile water followed by centrifugation of the extracts in a bench-top Eppendorf centrifuge for five minutes at 16.1 k rcf (13.2 k rpm). Virus captured on the TEM grids was stained with 1% phosphotungstate (PTA) solution. The grids were examined in a Hitachi H-7700 Electron Microscope at the Electron and Confocal Microscope Unit, Beltsville Agricultural Research Center.

## Results

### Nucleotide sequence and genome organization

Alfalfa plants collected in Sudan and used in this work exhibited chlorosis and stunting. Upon arrival, the samples were partially deteriorated; visual signs and symptoms for identification of any possible diseases were not clear enough to be recognized. Preliminary rounds of RT-PCR (not shown) with total RNAs extracted from the tissues suggested that plant sample designated 98.3A might be infected with several viral diseases that commonly occur in alfalfa. Total RNA from this sample was subjected to HTS to detect all potentially present viruses in an unbiased manner.

Raw data reads were assembled into contigs and searched against a custom virus database by the translated BLAST tool (BLASTX) [12]. Several essentially complete viral genomes were discovered in the sequenced sample: a novel flexivirus with a highest bit score for shallot virus X (ShVX), a virus with ~98% identity to peanut stunt virus (PSV, genus *Cucumovirus*, family *Bromoviridae*), and a virus with 90–97% identity to alfalfa enamovirus-1 (AEV-1, tentative member of the family *Luteoviridae*), [7]. To confirm the identity of the sequenced sample as *Medicago sativa* species, all host transcripts were assembled into a separate dataset and mapped onto the reference genome of cultivated alfalfa (inbred lines of cultivated alfalfa at the diploid level; CADL, 2*n* = 2*x* = 16; communicated to LGN by Deborah Samac and Nevin D. Young). The high percent identity (93–100%) confirmed the sample as alfalfa.

While PSV and AEV-1 have been reported in alfalfa [7, 13], AVS is a new, previously undescribed species. A complete nucleotide sequence of the viral genome obtained by *de-novo* assembly of HTS-generated reads, 5’RACE and sequencing of the RT-PCR-amplified 3’terminus, consisted of 8,349 nucleotides excluding the 3’ poly(A) tail. NCBI BLAST searches revealed that the virus has its greatest sequence identity with members of the family *Alphaflexiviridae*, genus *Allexivirus*. On the nucleotide level, the species most similar to the AVS were isolates A and B (54.6% and 53.7% identities, respectively) of unclassified arachis pintoi virus [14]; an unassigned species within the family *Alphaflexiviridae*, blackberry virus E (BVE) [15], (50.6%); and the type member of the genus *Allexivirus*, ShVX [16], (49.7%). Nucleotide identities were obtained by sequence-based virus classification tool, Pairwise Sequence Comparison (PASC) [17].

**Fig 1:**
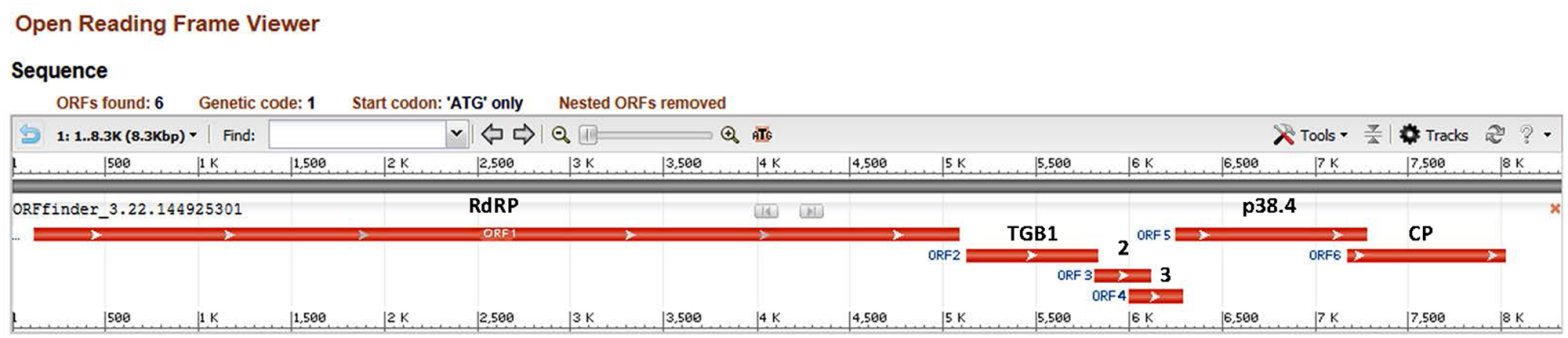
Adjusted open reading frame finder output (ORFfinder, NCBI, https://www.ncbi.nlm.nih.gov/orffinder/) showing genome organization of alfalfa virus S. ORF1, encoding RNA-dependent RNA polymerase (RdRp); ORF 2,3, and 4, encoding triple gene block proteins (TGB) 1, 2 and TGB3-like protein, respectively; ORF5, encoding p38.4, an unknown protein; ORF6, encoding AVS coat protein (CP).

The complete AVS genome (GenBank accession № KY696659) contains six computationally predicted open reading frames (ORF), encoding the following putative proteins (Fig 1): ORF1, viral replication protein (RNA-dependent RNA polymerase, RdRp, nt 119–5092, 1658 aa); ORF2, triple gene block protein 1 (TGB1, nt 5123–5839, 239 aa); ORF3, TGB2 (nt 5817–6119, 101 aa); ORF4, TGB3-like protein starting with the canonical AUG initiation codon (nt 5992–6288, 99aa); ORF5, an unknown 38.4 kDa protein resembling the serine-rich 40 kDa protein characteristic for allexiviruses [16,18,19], (nt 6245–7279, 345 aa) and ORF6, coat protein (CP, nt 7170–8027, 286 aa). It appears that AVS lacks a clear 3’ proximal ORF that is typical for allexiviruses and encodes a nucleic acid-binding protein [16,18,19]. The 5’untranslated region of AVS includes 118 nucleotides (1–118), and the 3’ end consists of 322 nt (8028–8349).

ORF1 of AVS (RdRp) has four conserved domains: viral methyltransferase, (accession pfam01660, nt 41–333, E-value 6.24e-72), 2OG-Fe(II) oxygenase (pfam13532, nt 604–721, E-value 2.42e-12), viral helicase 1, (pfam01443, nt 901–1134, E-value 1.88e-56) and RNA dependent RNA polymerase (pfam00978, nt 1308–1499, E-value 1.22e-06). ORF1 also has domain hits with alkylated DNA repair dioxygenase AlkB (accession COG3145, nt 604–721, E-value 4.82e-06), prolyl 4-hydroxylase alpha subunit homologues (smart00702, nt 620–720, E-value 2.72e-03), and ATP-dependent exoDNAse (COG0507, nt 1096–1134, E-value 5.30e-03).

ORF4 is predicted to encode a TGB3-*like* protein since recognized allexiviruses, unlike AVS, lack canonical TGB3 ORF with the AUG initiation codon [16,20]. The TGB3-like protein has a BLASTp hit with “7kD viral coat protein domain” from carlaviruses and potexviruses (pfam02495, E-value 7.91e-04), with the TGB3 of the Escobaria virus, an unclassified member of the family *Alphaflexiviridae* (GenBank: AHB87054.1) and with the 8kDa protein of unclassified white ash mosaic virus (GenBank: ADI70511.1) [21].

ORF 5 (encoding unknown 38.4kDA protein) has 36% identity scores (query coverage 91%, E-value 1e-56) with the 40kDa protein of arachis pintoi virus, unclassified member of the family *Alphaflexiviridae* (accession YP_009328895.1), 36% identity (75% coverage, E-value 6e-44) with *Garlic virus A*, member of the genus *Allexivirus* (accession AGC09138.1) and 35% identity (75% coverage, E-value 2e-41) with p42 of ShVX, a type member of the genus *Allexivirus* (NP_620651.1). This polypeptide is a distinctive feature of all known allexiviruses [19,20] and is presumably involved in the virion assembly process [16].

ORF 6 (coat protein), has 46% identity (83% coverage, E-value 8e-68) with the CP of ShVX (GenBank accession ACF37244.1), 49% identity (coverage 77%, E-value2e-67) with CP of arachis pintoi virus A (APG31859.1, and 51% identity (coverage 75%, E-value 3e-68) with shallot mite-borne latent virus, unclassified allexivirus possibly synonymous with ShVX [22], (GenBank: ACF37242.1). The first AUG codon of ORF 6 is at position 7170–7172 nt, which is 107 nt 5’ of the ORF5 termination codon, and the presumptive ORF 6 ends at the position 8027. There are four other possible initiation codons downstream of the first AUG, including two that are positioned 3’ of the ORF5 termination codon: nt 7209–7211, nt 7269–7271, nt 7290–7292, and nt 7308–7310. Although leaky scanning expression from these four downstream initiation sites is possible, they appear to be in a relatively weak context compared to the first initiation codon [23]. Overlapping ORFs are common among positive strand RNA plant viral genomes [24].

### Phylogenetic analysis

**Fig 2:**
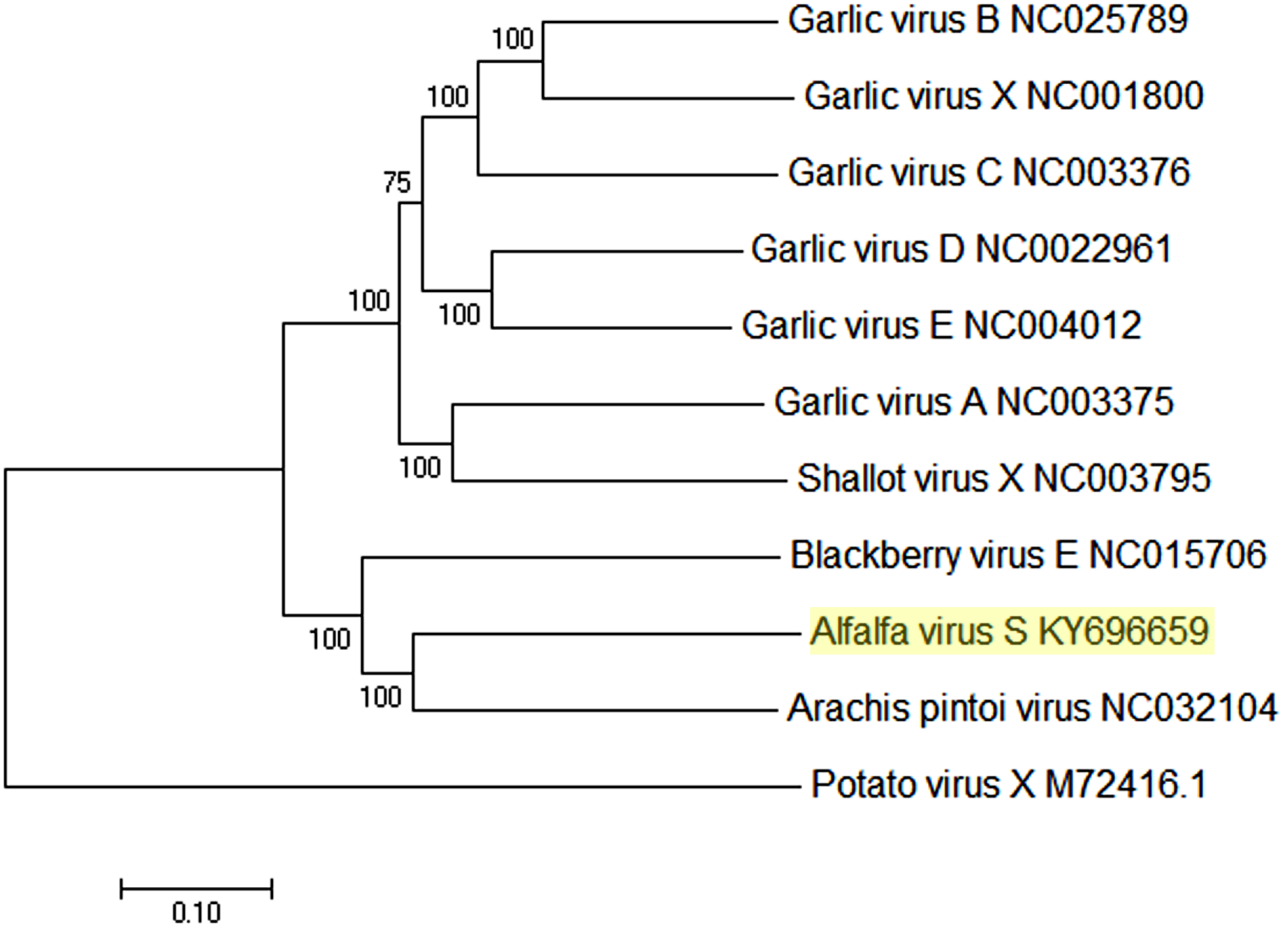
Phylogenetic relationship between alfalfa virus S (highlighted), classified allexiviruses and unassigned members of the family *Alphaflexiviridae*. The tree was built based on the available complete nucleotide sequences using MEGA 7 software (version 7.0.21) [25] and the Neighbor-Joining method. The optimal tree with the sum of branch length = 3.10976014 is shown. The percentage of replicate trees in which the associated taxa clustered together in the bootstrap test (1000 replicates) are shown next to the branches.

A phylogenetic tree was generated with the complete nucleotide sequences of all currently known allexiviruses, closely related unclassified members of the family *Alphaflexiviridae* and the AVS genome (Fig 2). AVS grouped with arachis pintoi virus A (54.6% identity vs AVS), branching out of the larger BVE group (50.6% identity to AVS), which is directly connected to the cluster of established *Allexivirus* species.

### Confirmation of the identity and specific detection of the virus

**Fig 3:**
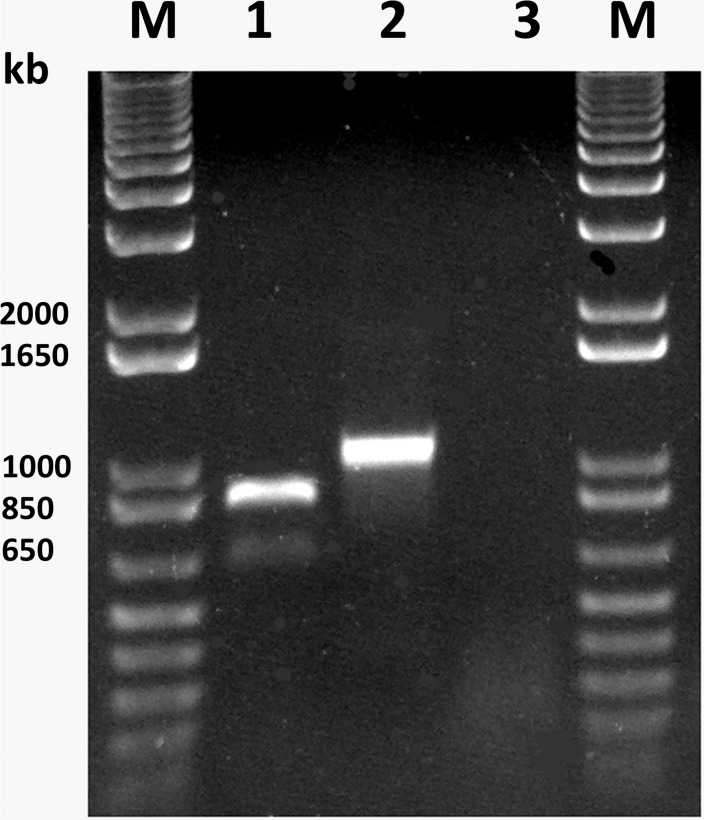
RT-PCR with total RNA extracted from the original alfalfa sample 98.3A. Lane 1, amplification with primers LN409/LN411, specific for the AVS coat protein. Lane 2, amplification with primers LN413/LN414, specific for the p38.4 protein. Lane 3, primers LN409/LN411 specific for the AVS coat protein were used, with RNA extracted from a healthy alfalfa plant. M, 1kb plus DNA ladder (ThermoFisher Scientific).

A complete genome of the AVS obtained via RNA-sequencing was used to design several sets of primers for RT-PCR to confirm the virus identity and accuracy of the nucleotide sequence. Using primer pairs for CP ORF and p38.4 ORF (Table 1) with total RNA extracted from the original infected sample 98.3A, respective virus genes were successfully amplified (Fig 3), cloned into pCRII-TOPO vector (ThermoFisher Scientific) and sequenced. Sequence data completely matched the HTS results thus validating the quality of the digital assembly and the presence of the predicted virus.

**Fig 4:**
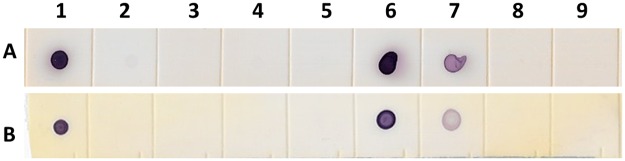
Dot blot hybridization assay with DIG-labeled RNA probes. RNA probes derived from the AVS CP. (A) and p38.4 (B) ORFs. Total RNA was extracted from the following alfalfa samples: 1, 98.3A; 2, 89.2AS; 3, 89.3H; 4, 89.1H; 5, 89.1AS; 6, 98.1A; 7, 98.3A (0); 8, 98.2RR and 9, 97.2A.

To further support the existence of the pathogen in the defined molecular form, DIG-labeled RNA probes were generated from the cloned p38.4 and CP cDNAs and used in dot blot hybridization assays with RNA extracted from infected alfalfa tissues. Both RNA probes successfully detected virus in the infected samples 98.3A, 98.1A and 98.3A(0), which are different alfalfa plants belonging to the same cultivar (Fig 4).

**Fig 5:**
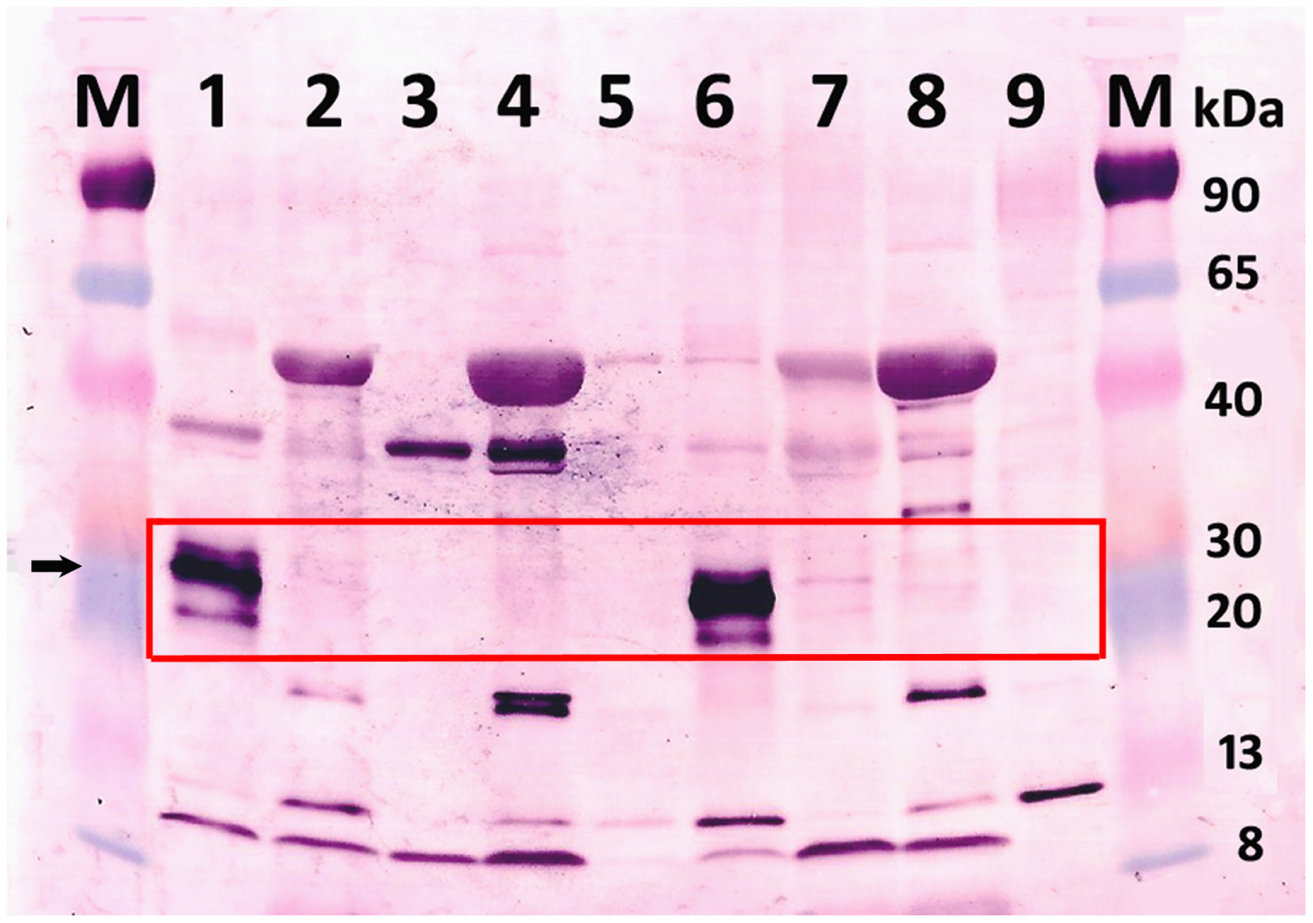
Western blot analysis of the alfalfa samples. Membranes were probed with antibody produced against the peptide CP1. M, ColorBurst electrophoresis marker (Sigma). Lane 1 to 9: alfalfa samples 98.3A (hybridization-positive), 89.2AS, 89.3H, 89.1H, 89.1AS, 98.1A (hybridization-positive), 98.3A(0) (hybridization-positive), 98.2RR and 97.2A, respectively. Arrow indicates bands specific to the AVS CP.

Molecular data were also supported by immunological detection of the virus: polyclonal antibodies, produced against the peptide CP1 within the AVS CP specifically reacted with alfalfa samples 98.3A, 98.1A and less obviously with the sample 98.3A(0) in WB assay (Fig 5). The CP migration in the 10–20% SDS gel slightly differed from the predicted protein size (~29 kDa vs 31kDA, respectively). The faster migration can probably be explained by atypical protein-SDS binding, albeit leaky scanning and initiation of the CP translation from alternative downstream codons remains a possibility. The same three samples were tested positive in dot blot hybridization assays with the RNA probes to AVS CP and p38.4 (Fig 4). Antibodies to peptide CP2 reacted less specifically, while Abs to peptide CP3 did not react at all (not shown). Thus, western blot analysis suggested the presence of antigenicity near the N-terminal region of the AVS CP.

### Transmission electron microscopy

**Fig 6:**
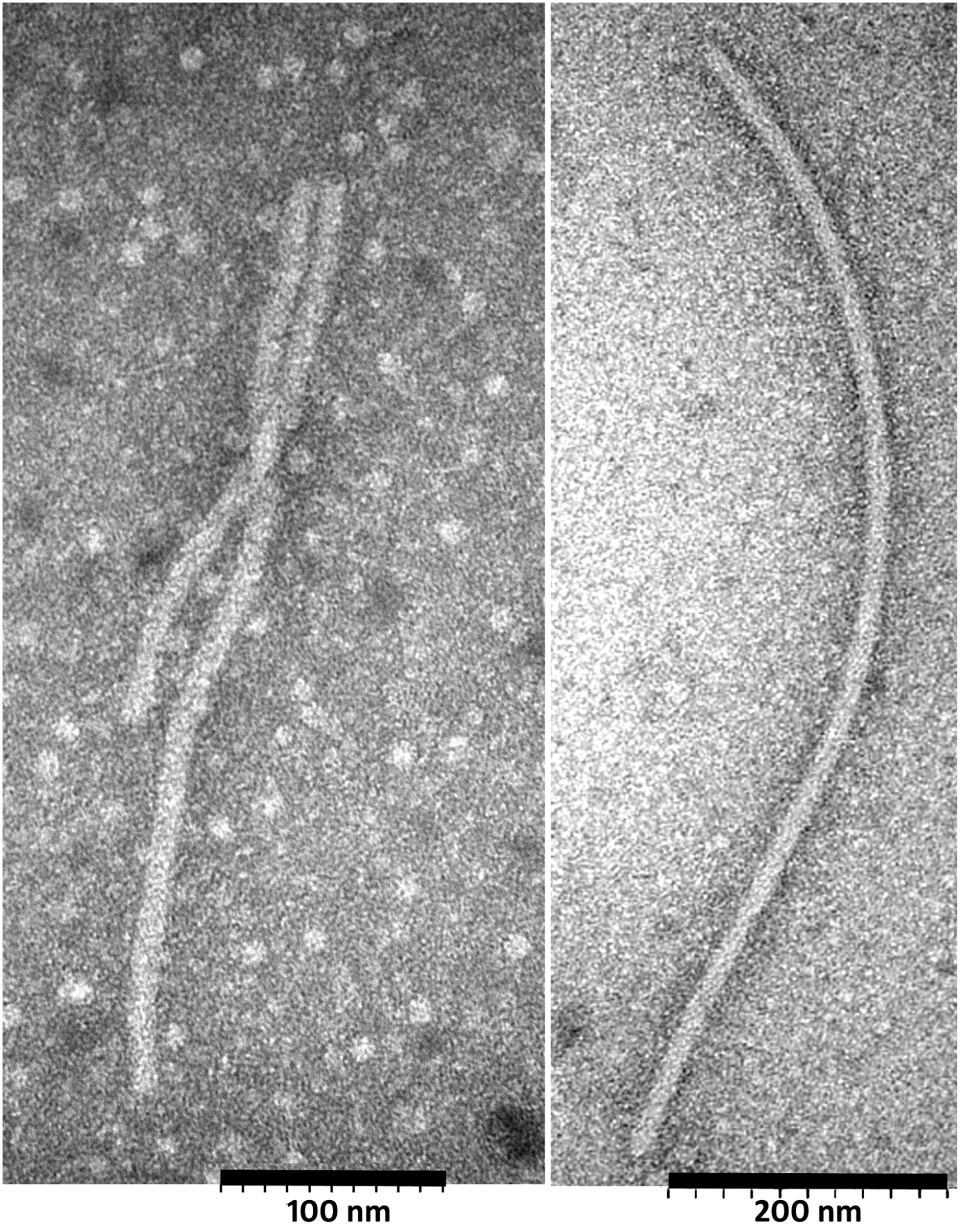
Electron micrographs of viral particles observed in the AVS-infected alfalfa samples 98.3A, 98.1A and 98.3A(0). Scale bars represent 100 nm and 200 nm (left panel and right panel, respectively).

The original sequenced sample 98.3A used for HTS analysis and also two more samples, 98. 1A and 98.3A (0), that were later confirmed to be positive for AVS by dot blot hybridization and WB, were examined in a Hitachi H-7700 Electron Microscope. Filamentous virus particles about 600–800 nm in length and 13–16 nm in diameter were observed in the extracts from all tested samples (Fig 6). Shorter and longer filaments possibly representing broken particles and end-to-end aggregation of two virions, respectively, were also observed (not shown).

## Discussion

As of today, the natural host range of all ICTV-classified allexiviruses is thought to be restricted to *Allium* spp. of monocotyledonous flowering plants, although some allexiviruses will induce local lesion on *Chenopoidium* species [16,18,19]. Here we report on the discovery, characterization and sensitive detection of a novel viral pathogen, resembling allexiviruses, in dicotyledonous species *Medicago sativa*, legume family *Fabaceae*. The virus, tentatively named “alfalfa virus S” (AVS), is an unusual pathogen most closely related to arachis pintoi virus and BVE, which are putative and unclassified members of the family *Alphaflexiviridae*, respectively, and to several members of the genus *Allexivirus*. Even though the identity level between AVS and accepted allexiviruses is rather low, it has some of the characteristic features only found in allexiviruses, such as a serine-rich p38.4 protein of unknown function, as well as its general genome organization. Phylogenetic analyses clearly placed AVS together with arachis pintoi virus and BVE in a distinct cluster associated with the identified allexiviruses (Fig 2). However, unlike arachis pintoi virus (lacks TGB3) and BVE (lacks an AUG initiation codon of TGB3), the putative TGB3-like protein of the AVS has an AUG initiation codon, suggesting the functional importance of TGB3 rather than any alternative strategy for the viral cell-to-cell movement [15]. Similarly to arachis pintoi virus and BVE, no sequences homologous to the 3’ proximal nucleic acid-binding protein of allexiviruses were identified in the assembled genome of AVS, perhaps suggesting the need for a new genus, accommodating these viruses.

The identity of the virus was confirmed by RT-PCR with primers derived from the HTS-generated sequence, dot blot hybridization with DIG-labeled virus-specific RNA probe, and WB analysis with antibodies produced against a viral CP peptide. Each of these methods can be of practical use for accurate diagnosis, identification and control of AVS in single infection or in complex multiviral epidemics of alfalfa. TEM observations have shown a presence in the infected tissues of filamentous virions similar to allexiviruses in their length and appearance.

We conclude that AVS represents a unique virus species with a general resemblance to the members of the genus *Allexivirus* and a more evident relation to the unclassified arachis pintoi virus and unassigned alphaflexivirus BVE. However, a low level of the whole genome homology with the most closely related viral species (49%-54%) indicates that AVS is a member of a novel, previously undescribed genus.

## References

[1] Li X, Brummer EC. Applied genetics and genomics in alfalfa breeding. Agronomy. 2012; 2:40–61

[2] Monteros MJ, Bouton JH. The future of alfalfa and forage crops In: Proceedings, Western Alfalfa & Forage Conference, December 2–4, Reno, Nevada, 2009.

[3] Bailiss KW, Ollennu LAA. Effect of alfalfa mosaic virus isolates on forage yield of lucerne (*Medicago sativa*) in Britain. Plant Pathology.1986; 35:162–168.

[4] Samac DA, L. H. Rhodes LH, W. O. Lamp WO, Diseases of alfalfa (*Medicago sativa*). APS publications. The American Phytopathological Society 2017 http://www.apsnet.org/publications/commonnames/Pages/Alfalfa.aspx

[5] Frate CA, Davis RM. Irrigated alfalfa management for Mediterranean and desert zones. University of California, Division of Agriculture and Natural Resourses 2008; Publication 8296, chapter 10.

[6] van Leur JAG, Kumari SG. A survey of lucerne in northern New South Wales for viruses of importance to the winter legume industry. Australasian Plant Pathol. 2011; 40:180–186.

[7] Bejerman N, Giolitti F, Trucco V, de Breuil S, Dietzgen RG, Lenardon S. Complete genome sequence of a new enamovirus from Argentina infecting alfalfa plants showing dwarfism symptoms. Arch Virol. 2016; 161:2029–32. 10.1007/s00705-016-2854-3 27068164

[8] Bejerman N, Nome C, Giolitti F, Kitajima E, de Breuil S, Perez Fernandez J, et al First report of a Rhabdovirus infecting alfalfa in Argentina. Plant Dis. 2011; 95:771. 10.1094/PDIS-10-10-0764 30731911

[9] Massumi H, Maddahian M, Heydarnejad J, Hosseini Pour A Farahmand A. Incidence of viruses infecting alfalfa in the southeast and central regions of Iran. Journal of Agricultural Science and Technology. 2012; 14:1141–1148.

[10] van Miltenburg R, Rüger B, Grünewald-Janho S, Leons M, Schrӧder C. The DIG system user’s guide for filter hybridization. 1995; Boehringer Mannheim GmbH, 100 p.

[11] Nemchinov LG, Natilla A. Transient expression of the ectodomain of matrix protein 2 (M2e) of avian influenza A virus in plants. Protein Expression and Purification. 2007; 56:153–9 17644356 10.1016/j.pep.2007.05.015

[12] Altschul SF, Gish W, Miller W, Myers EW, Lipman DJ. Basic local alignment search tool. J. Mol. Biol. 1990; 215:403–410. 10.1016/S0022-2836(05)80360-2 2231712

[13] Bananej K, Hajimorad MR, Roossinck MJ, Shahraeen N. Identification and characterization of peanut stunt cucumovirus from naturally infected alfalfa in Iran. Plant Pathology. 1998; 47:355–361.

[14] Sánchez PAG. Mesa HJ, Montoya MM. Next generation sequence analysis of the forage peanut (*Arachis pintoi*) virome Revista Facultad Nacional de Agronomía. 2016; 10.15446/rfna.v69n2.59133.

[15] Sabanadzovic S, Abou Ghanem-Sabanadzovic N, Tzanetakis IE. Blackberry virus E: an unusual flexivirus. Arch Virol. 2011; 156:1665–9. 10.1007/s00705-011-1015-y 21656298

[16] Zavriev SK. *Allexivirus*. Encyclopedia of Virology, 5 vols (Mahy BWJ and Van Regenmortel MHV, Editors). 2008; pp. 96–98 Oxford: Elsevier.

[17] Bao Y, Chetvernin V, Tatusova T. Improvements to pairwise sequence comparison (PASC): a genome-based web tool for virus classification. Arch Virol. 2014;159:3293–3304. 10.1007/s00705-014-2197-x 25119676 PMC4221606

[18] Adams MJ, Antoniw JF, Bar-Joseph M, Brunt AA, Candresse T, Foster GD, et al The new plant virus family Flexiviridae and assessment of molecular criteria for species demarcation. Arch Virol. 2004; 149:1045–60. 10.1007/s00705-004-0304-0 15098118

[19] Vaira AM, Kreuze J, Hammond J, Pearson MN, Martelli GP, Adams MJ, et al Alphaflexiviridae In: King Andrew M.Q., Adams Michael J., Carstens Eric B., and Lefkowitz Elliot J., editors, Virus Taxonomy. Oxford: Elsevier, 2011, pp. 904–919.

[20] Lezzhov AA, Gushchin VA, Lazareva EA, Vishnichenko VK, Morozov SY, Solovyev AG. Translation of the shallot virus X TGB3 gene depends on non-AUG initiation and leaky scanning. J Gen Virol. 2015; 96:3159–64. 10.1099/jgv.0.000248 26296665

[21] Machado-Caballero JE, Lockhart BE, Mason SL, Mollov D. Identification, Transmission, and partial characterization of a previously undescribed flexivirus causing a mosaic disease of ash (*Fraxinus* spp.) in the USA. Plant Health Progress, Plant Management Network. 2013; 10.1094/PHP-2013-0509-01-RS

[22] Perez-Egusquiza Z, Ward LI, Clover GRG, J. D. Fletcher JD, van der Vlugt RAA. First report of *Shallot virus X* in shallot in New Zealand. Plant Pathology. 2009; 58, 407.

[23] Kozak M.Pushing the limits of the scanning mechanism for initiation of translation. Gene. 2002; 299:1–34. 12459250 10.1016/S0378-1119(02)01056-9 PMC7126118

[24] Dreher TW, Miller WA. Translational control in positive strand RNA plant viruses. Virology. 2006;344:185–97. 10.1016/j.virol.2005.09.031 16364749 PMC1847782

[25] Kumar S, Stecher G, Tamura K. MEGA7: Molecular Evolutionary Genetics Analysis Version 7.0 for Bigger Datasets. Mol Biol Evol. 2016; 33:1870–4. 10.1093/molbev/msw054 27004904 PMC8210823

